# Efficacy of conjunctival flap surgery for deep corneal ulcers

**DOI:** 10.22336/rjo.2021.33

**Published:** 2021

**Authors:** Alina-Cristina Stamate, Călin Petru Tătaru, Mihail Zemba

**Affiliations:** * Department of Ophthalmology, “Carol Davila” University of Medicine and Pharmacy, Bucharest, Romania; ** Arena Med Clinic, Bucharest, Romania; *** Clinical Hospital of Ophthalmologic Emergencies, Bucharest, Romania; **** Department of Ophthalmology, “Dr. Carol Davila” Central Military Emergency University Hospital, Bucharest, Romania

**Keywords:** deep corneal ulcers, corneal perforations, conjunctival flap surgery, emergency, ocular surface integrity

## Abstract

**Aim:** To evaluate the clinical efficacy of a selective, partial, pedicle conjunctival flap in the treatment of deep corneal ulcers with or without perforation, resistant to medical treatment.

**Method:** This interventional self-controlled retrospective study included 31 eyes of 31 patients with deep corneal ulcers who underwent conjunctival flap surgery in a tertiary eye care unit between 2017 and 2019. Of these, 9 eyes exhibited corneal perforation. The follow-up period was 12 months. The primary outcome measures were restoring ocular surface integrity and secondary outcome measures were improvement in visual acuity and postoperative complications encountered.

**Results:** Out of the total of 31 patients, 17 patients (55%) were males and 14 patients (45%) were females. The mean age was 56.03 ± 15.46 years. The mean disease duration was 64.10 ± 35.01 days, the mean diameter of the ulcer was 3.61 ± 1.02 mm and the mean depth of the ulcer was 70.65 ± 20.28% of the thickness of the cornea. The etiology was extensive and the corneal ulcers were categorized as infectious (12), noninfectious (16), and unknown (3). An anatomic cure was obtained in 29 (94%) of 31 eyes. Conjunctival flap surgery was unsuccessful in 2 eyes that required evisceration. The postoperative visual acuity (BCVA) improved in 13 (42%) of the 31 eyes, decreased in 9 eyes (29%) and remained unchanged in 9 eyes (29%). The most frequent complications after conjunctival flap surgery were pseudopterygium, cataract and corneal opacity and less frequent complications were glaucoma, astigmatism, flap retraction, corneal perforation and endophthalmitis.

**Conclusions:** Conjunctival flap surgery can successfully treat refractory deep corneal ulcers. It can restore ocular surface integrity and provide metabolic and mechanical support for corneal healing. Also, it can avoid emergency penetrating keratoplasty or create appropriate conditions for a future optic keratoplasty.

## Introduction

Corneal diseases are a major cause of monocular blindness worldwide and can be considered a real silent epidemic. The etiology of corneal blindness is complicated and includes a large variety of inflammatory and infectious diseases that can cause corneal scarring and are responsible for functional blindness. The prevalence of these pathologies varies from one country to another, and even from one population to another, and often tend to be underreported. It is estimated that 1.5 to 2 million cases of corneal blindness are newly diagnosed annually due to ocular trauma and infectious keratitis in developing countries [**1**]. 

Despite the antimicrobial treatments available for most of the microorganisms involved in the pathogenesis of infectious keratitis, the clinical results are often disappointing. The strategies required to reduce the complications associated with this pathology should be multistage and should include improving early and accurate diagnostics techniques, developing more effective antimicrobial agents to overcome drug resistance and prevention of corneal ulceration [**2**]. 

Corneal ulcerations, and more specifically corneal perforations, are a real concern and represent an ocular emergency that requires prompt intervention and treatment. Although various medical treatments are available, such severe cases are only curable with surgical treatment. The short-term goals of the surgical intervention should be to seal the ulcerated or perforated area and to provide tectonic support, in order to avoid serious ocular morbidities that can eventually lead to evisceration or enucleation [**3**]. 

Conjunctival flap surgery is a simple, effective, and affordable treatment for corneal ocular surface disease unresponsive to medical treatment. It can assure a stable ocular surface and repress local inflammation [**4**]. Although its use has deeply decreased in developed countries after the evolution of therapeutic penetrating keratoplasty, amniotic membrane transplants and epithelial transplants techniques, it can still be of use in selected cases or in the absence of donor tissue or surgical instruments or lack of surgical experience for the forementioned techniques [**5**-**7**]. Conjunctival flap surgery can also achieve several other secondary effects: pain relief, reduced topical drugs, improved aesthetic aspect and option to invasive surgery [**5**,**8**,**9**]. In some situations, it can also be used as temporizing method for a future corneal transplant [**10**].

## Material and methods

***Study design and patients***

This was an interventional self-controlled retrospective study. The study was approved by the ethics committee of “Dr. Carol Davila” Central Military Emergency University Hospital (Bucharest, Romania) and was in concordance with the tenets of the Declaration of Helsinki. A total of 31 patients who underwent conjunctival flap surgery for deep corneal ulcers between January 2017 and December 2019 at the hospital were enrolled. 

 Medical records were reviewed and data were collected from the patients, including age, gender, living environment, duration of onset, cause of ulceration, uncorrected visual acuity (UCVA) and best-corrected visual acuity (BCVA), intraocular pressure (IOP) and predisposing associated factors (i.e., past or current topical or systemic treatment, systemic disorders, contact lens wear, ocular trauma), and all the information was used only for research purposes. 

The indications for surgical treatment were deep corneal ulcer (>50% loss of stroma), descemetocele or corneal perforation and medical treatment failure (no improvement after 2 weeks of intensive medical treatment). The patients who met these criteria were considered eligible for surgery. 

***Preoperative evaluation***

All the patients underwent a complete ophthalmic evaluation. Symptoms such as pain, redness, photophobia, watering, and discharge were registered. The evaluation focused on visual acuity testing (UCVA and BCVA) and slit lamp evaluation of the conjunctiva, cornea, anterior chamber, iris, pupil, and lens. Neovascularization, synechiae, hypopyon, ulcer size and site, ulcer staining and corneal infiltrates were also noted. 

***Surgical technique***

All the surgeries in the study were performed by the same surgeon. Surgery was conducted in a sterile surgical theatre with the patient seated in supine position under the operating microscope. Retrobulbar anesthesia was administered to the affected eye and povidone iodine was applied with a cotton ball to sterilize the tegument around the eye. After draping of the eye, an eye spectrum was inserted. The corneal epithelium and necrotic tissue from the ulceration site or within 1 mm of the ulcer margin were removed with a surgical blade. A marker pen was used to mark the margin of the conjunctival segment that offered the best blood supply to the corneal ulcer, from the quadrant most near to the ulcer. After subconjunctival injection of balanced 2% lidocaine with 1:100.000 epinephrine to separate the conjunctiva from the underlying Tenon’s capsule, a tongue-shaped incision was performed. The created conjunctival flap was mobilized to cover the corneal ulcer and secured to the episclera opposite the site of incision, with interrupted 10-0 nylon sutures. At the end of the surgery, topical antibiotics were applied and the eye was dressed. The sutures were removed after 2 weeks.

***Postoperative evaluation***

**Fig. 1:**
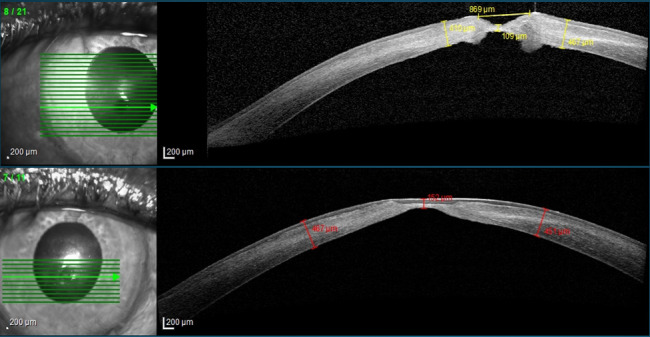
AS-OCT evaluation of corneal ulcer: preoperative (superior figure) and postoperative (inferior figure). The conjunctival flap healed the corneal tissue. Postoperatively, the involved cornea was thickened, semitransparent, and highly reflective

The follow-up period was 12 months. The postoperative evaluation included visual acuity testing (UCVA and BCVA), IOP and slit lamp evaluation of the cornea (i.e., ulceration, edema, and neovascularization) and anterior chamber (i.e., inflammation, synechiae, hypopyon). Fluorescein staining was used to detect corneal epithelial defects and anterior segment optical coherence tomography (AS-OCT) was performed to examine the corneal tissue adjacent to the conjunctival flap (**Fig. 1**).

***Statistical analysis***

The data were processed using SPSS software version 22. Both descriptive and inferential statis¬tical analyses were performed. The descriptive statistics was used to express data in terms of frequency and percentage. Statistical data were expressed in terms of means ± standard deviations (mean ± SD). Using the number of postoperative complications and secondary surgical interventions as dependent variables and depth of the ulcer as independent variable, inferential statistics were used to investigate whether the depth of the ulcer influenced the occurrence of complications and ulterior surgeries after the initial conjunctival flap surgery. A repeated measure ANOVA was used to determine whether there was a change in BCVA preoperatively and postoperatively. P value < 0.05 was considered statistically significant.

## Results

***Patient characteristics***

A total of 31 eyes from 31 patients were included in the study. Out of the total of 31 patients, 17 patients (55%) were males and 14 patients (45%) were females. The mean age was 56.03 ± 15.46 years. The mean disease duration was 64.10 ± 35.01 days, the mean diameter of the ulcer was 3.61 ± 1.02 mm and the mean depth of the ulcer was 70.65 ± 20.28% of the thickness of the cornea (9 patients exhibited corneal perforation). The causes of corneal ulcers were categorized as infectious (12), noninfectious (16), and unknown (3). The causes of infectious ulcers included bacterial (6), viral (4) and fungal (2) keratitis. The causes of noninfectious ulcers included bullous keratopathy (4), neurotrophic keratitis (3), pterygium surgery (4), foreign body (2), rheumatoid arthritis (2), and atopic keratoconjunctivitis (1). The etiology of the corneal ulcer was unknown in 3 eyes, 2 of which had a central perforation. 

***Patient outcomes***

***1. Disease cure:*** An anatomic cure was obtained in 29 (94%) of the 31 eyes. Conjunctival flap surgery was unsuccessful in 2 cases. The first case was a central corneal ulcer due to viral keratitis, which suffered flap perforation with secondary corneal perforation and required another flap surgery. Again, the flap retracted and the eye eventually developed endophthalmitis and needed evisceration. The second case was one of the ulcers of unknown etiology with a central corneal perforation, which also suffered flap perforation, underwent another flap surgery, and later developed endophthalmitis and needed evisceration. Of the initial 9 corneal perforations, 8 recovered and regained anatomical integrity. 11 patients (35%) underwent only one surgical intervention and 20 of them (65%) required further surgeries such as removal of conjunctival flap, repeating flap surgery, cataract surgery, penetrating keratoplasty and evisceration. There was no significant statistical correlation between the depth of the ulcer and the number of secondary surgical interventions (p=0.215).

**Fig. 2:**
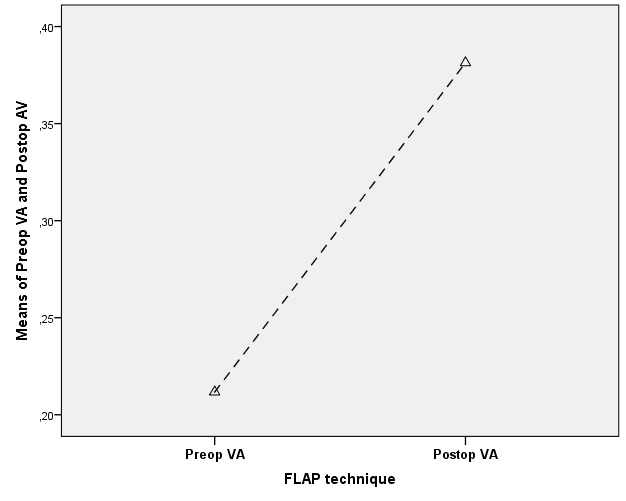
Comparison between preoperative and postoperative visual acuity

***2. Visual prognosis:*** The postoperative visual acuity (BCVA) improved in 13 of the 31 cases (42%), decreased in 9 cases (29%) and remained unchanged in 9 cases (29%). A repeated measure ANOVA was performed in order to determine whether there was a change in the visual acuity before and after conjunctival flap surgery. According to the Bonferroni correction test, there was a statistically significant change (p=0.003) and most patients had an improvement in visual acuity after the surgery (0.38 vs. 0.21) (**Fig. 2**).

***3. Postoperative complications:*** The most frequent complications after conjunctival flap surgery were pseudopterygium, cataract and corneal opacity and less frequent complications were glaucoma, astigmatism, flap retraction, corneal perforation and endophthalmitis. There was no significant statistical correlation between the depth of the ulcer and the number of postoperative complications (p=0.215).

## Discussion

Conjunctival flap surgery proved over time to be effective in curing corneal ulcers. The therapeutic effects of this type of intervention are possibly promoted by several mechanisms. First, the flap prevents tears, proteolytic enzymes and proinflammatory mediators from reaching the corneal ulcer and causing further stromal ulceration [**10**]. Secondly, the rich supply of blood vessels and lymphatics offers nutrients, such as cellular components and growth factors, that increase the resistance to infection, and anticollagenolytic substances that inhibit stromal lysis [**10**-**12**]. 

In the present study, the patients underwent selective, partial, pedicle conjunctival surgery and the flap was mobilized only on the ulcerated cornea, as opposed to the total conjunctival flap described by Gundersen, which covered the entire cornea. The benefit of this technique was that it offered an alternative to Gundersen’s flap and avoided its complications.

An anatomic cure was obtained in 29 of 31 eyes in our study (94%), and 2 eyes eventually required evisceration due to complications. Of the initial 9 corneal perforations included in the study, 8 recovered and regained anatomical integrity. Our results were similar to the ones obtained by Zhou et al. in their study, which recorded a cure rate of 93% and also included refractory corneal ulcers of various etiologies [**13**].

Most of the patients in the study required conjunctival flap surgery to prevent or cure corneal perforation and to preserve the ocular globe, rather than improve visual acuity. Despite this fact, the postoperative visual acuity (BCVA) improved in 13 of the 31 cases (42%), decreased in 9 cases (29%) and remained unchanged in 9 cases (29%). In such conditions, the decreased or the unchanged postoperative visual acuity did not reflect that conjunctival flap surgery was unsuccessful. 

11 patients (35%) underwent only one surgical intervention and 20 of them (65%) required further surgeries such as removal of conjunctival flap, repeating flap surgery, cataract surgery, penetrating keratoplasty and evisceration. The number of ulterior surgeries did not emphasize a failure of the initial conjunctival flap surgery, but rather the severity of the disease. 

The limit of this study was the small number of cases, and the fact that the etiology was so vast, thus preventing the drawing of conclusions regarding the efficacy of pedicle conjunctival flap surgery in certain etiologies. Further studies with larger case series are required to verify this. 

## Conclusion

 In conclusion, conjunctival flap surgery can successfully treat refractory deep corneal ulcers. It can restore ocular surface integrity and provide metabolic and mechanical support for corneal healing. Also, it can avoid emergency penetrating keratoplasty or create appropriate conditions for a future optic keratoplasty, which is especially important in countries that lack corneal tissue.

**Conflict of Interest statement**

Authors state no conflict of interest.

**Informed Consent and Human and Animal Rights statement**

Informed consent has been obtained from all individuals included in this study.

**Authorization for the use of human subjects**

Ethical approval: The research related to human use complies with all the relevant national regulations, institutional policies, is in accordance with the tenets of the Helsinki Declaration, and has been approved by the ethics committee of “Dr. Carol Davila” Central Military Emergency University Hospital (Bucharest, Romania).

**Acknowledgements**

None.

**Sources of Funding**

None.

**Disclosures**

None of the authors has any financial or proprietary interests to disclose.

## References

[1] Raj A, Bahadur H, Dhasmana R (2018). Outcome of therapeutic penetrating keratoplasty in advanced infectious keratitis. J Curr Ophthalmol.

[2] Austin A, Lietman T, Rose-Nussbaumer J (2017). Update on the Management of Infectious Keratitis. Ophthalmology.

[3] Bhatt P, Lim L, Ramaesh K (2007). Therapeutic deep lamellar keratoplasty for corneal perforations. Eye.

[4] Gundersen T, Pearlson HR (1969). Conjunctival flaps for corneal disease: their usefulness and complications. Trans Am Ophthalmol Soc.

[5] Lim LS, How AC, Ang LP, Tan DT (2009-08). Gundersen flaps in the management of ocular surface disease in an Asian population. Cornea.

[6] Zemba M, Stamate AC, Tataru CP, Branisteanu DC, Balta F (2020). Conjunctival flap surgery in the management of ocular surface disease (Review). Exp Ther Med.

[7] Stamate AC, Tătaru CP, Zemba M (2018). Emergency penetrating keratoplasty in corneal perforations. Rom J Ophthalmol.

[8] Sandinha T, Zaher SS, Roberts F, Devlin HC, Dhillon B, Ramaesh K (2006). Superior forniceal conjunctival advancement pedicles (SFCAP) in the management of acute and impending corneal perforations. Eye (Lond).

[9] Jhanji V, Young AL, Mehta JS, Sharma N, Agarwal T, Vajpayee RB (2011). Management of corneal perforation. Surv Ophthalmol.

[10] Stamate AC, Tătaru CP, Zemba M (2019). Update on surgical management of corneal ulceration and perforation. Rom J Ophthalmol.

[11] Sharma A, Mohan K, Sharma R, Nirankari VS (2014). Repositioning of pedicle conjunctival flap performed for refractory corneal ulcer. Middle East Afr J Ophthalmol.

[12] Abdulhalim BE, Wagih MM, Gad AA, Boghdadi G, Nagy RR (2015). Amniotic membrane graft to conjunctival flap in treatment of non-viral resistant infectious keratitis: a randomised clinical study. Br J Ophthalmol.

[13] Zhou Q, Long X, Zhu X (2010). Improved conjunctival transplantation for corneal ulcer. Zhong Nan Da Xue Xue Bao Yi Xue Ban.

